# The Acute Effect of Exercise on Arterial Stiffness in Healthy Subjects: A Meta-Analysis

**DOI:** 10.3390/jcm10020291

**Published:** 2021-01-14

**Authors:** Alicia Saz-Lara, Iván Cavero-Redondo, Celia Álvarez-Bueno, Blanca Notario-Pacheco, Marta Carolina Ruiz-Grao, Vicente Martínez-Vizcaíno

**Affiliations:** 1 Health and Social Research Center, Universidad de Castilla-La Mancha, 16171 Cuenca, Spain; Alicia.delSaz@uclm.es (A.S.-L.); celia.alvarezbueno@uclm.es (C.Á.-B.); Blanca.Notario@uclm.es (B.N.-P.); marta.ruiz@uclm.es (M.C.R.-G.); vicente.martinez@uclm.es (V.M.-V.); 2 Facultad de Ciencias de la Salud, Universidad Autónoma de Chile, 3460000 Talca, Chile

**Keywords:** arterial stiffness, pulse wave velocity, exercise, acute effect, healthy adults

## Abstract

Arterial stiffness has been shown to be a subclinical marker associated with cardiovascular disease. Meanwhile, long-term exercise has been demonstrated to reduce arterial stiffness, providing a decrease in cardiovascular risk. However, the acute effect of exercise on arterial stiffness is unclear. This systematic review and meta-analysis aimed to assess the acute effect of exercise interventions on arterial stiffness in healthy adults. We searched the Cochrane Central Register of Controlled Trials, MEDLINE (via Pubmed), Scopus, and Web of Science databases, from their inception to 30 June 2020. A meta-analysis was performed to evaluate the acute effect of exercise on arterial stiffness using random-effects models to calculate pooled effect size estimates and their corresponding 95% CI. Pulse wave velocity was measured as an arterial stiffness index. The 30 studies included in the meta-analysis showed that pulse wave velocity was not modified immediately after exercise (0 min post) (ES: 0.02; 95% CI: −0.22, 0.26), but subsequently decreased 30 min after exercise (ES: −0.27; 95% CI: −0.43, −0.12). Thereafter, pulse wave velocity increased to its initial value 24 h after exercise (ES: −0.07; 95% CI: −0.21, 0.07). Our results show that, although there is a significant reduction in pulse wave velocity 30 min after exercise, the levels of arterial stiffness return to their basal levels after 24 h. These findings could imply that, in order to achieve improvements in pulse wave velocity, exercise should be performed on a daily basis.

## 1. Introduction

Arterial stiffness (AS) is one of the first indicators detected in both functional and structural changes of the arterial wall and plays an important role in cardiovascular diseases (CVD) [1]. Previous evidence has shown that age, blood pressure, and unhealthy lifestyles such as inappropriate diet, lack of physical activity, and smoking [2] reduce the elasticity of the arteries [3]. AS is commonly measured by pulse wave velocity (PWv) [1], which is the non-invasive reference parameter of AS [4,5]. Furthermore, stiffening of elastic arteries (like the aorta and carotid artery) is related to different pathologies such as atherosclerosis, diabetes mellitus, dyslipidemia, and chronic kidney diseases, and it is therefore considered to be a predictor of CVD [6].

Moreover, current studies have observed that there is an inverse relationship between exercise and CVD [7], and this could be mediated by improved AS. This effect of exercise on AS could be related to a decrease in cardiovascular risk, estimated to be approximately 35 and 33% in CVD and all-cause mortality, respectively [7,8]. Although the long-term effects of exercise on AS have been demonstrated [9,10], the acute effect of different types of exercise on AS is unclear [11]. This could be due to the acute effect of exercise lasting only 24 h, probably because it is a functional and not a structural effect [12].

In 2018, a meta-analysis reported that acute aerobic exercise did not modify the central PWv, while resistance exercise increased the central PWv in healthy adults [11]. Additionally, numerous studies have shown conflicting results regarding the type of exercise and the length of its effectiveness when comparing the acute effects of different types of exercise on AS [13]. Currently, aerobic exercise is considered the most effective type of exercise to reduce AS, since most studies have reported a decrease in PWv at the central and peripheral levels in the first hour after exercise [14,15]. On the contrary, there are also studies suggesting no substantial changes in AS after the practice of acute exercise [16,17,18], and even that the AS increases immediately after the end of the exercise [19,20,21].

Due to the existing controversy about the acute effect of exercise in AS, it seems necessary to evaluate the possible beneficial or adverse acute effects of exercise in AS, a subclinical process underlying CVD. Therefore, the aims of this systematic review and meta-analysis were to (i) assess the acute effect of exercise interventions on central and peripheral AS in healthy adults; (ii) determine the evolution of the PWv by time periods from after exercise to 24 h later; (iii) compare the acute effects of different exercises (interval training, aerobic exercise, resistance training, stretching) on PWv.

## 2. Materials and Methods

This systematic review and meta-analysis is reported in accordance with the Preferred Reporting Items for Systematic Reviews and Meta-analyses (PRISMA) [22] and was conducted following the Cochrane Handbook for Systematic Reviews of Interventions recommendations [23]. This study was registered in PROSPERO (registration number: CRD42020206430).

### 2.1. Search Strategy

The systematic search of the studies was carried out through four databases—Cochrane Central Register of Controlled Trials, MEDLINE (via Pubmed), Scopus, and Web of Science—from their inception to 30 June 2020. To perform the search, the following keywords were used: “cardiovascular disease”, “cardiovascular risk”, “arterial stiffness”, “pulse wave velocity”, “PWv”, “physical activity”, “exercise”, “training”, “HIIT”, “interval training”, “intermittent exercise”, “continuous exercise”, “aerobic exercise”, “endurance training”, “resistance exercise”, “strength”, “stretching”, “stretches”, “acute”, “acute effect”, “immediate effect”, “healthy subjects”, “healthy participants”, “healthy people”, and “healthy adults”. The search strategy for the MEDLINE database is shown in Table S1. Furthermore, we searched the reference lists of included articles, as well as previous systematic reviews or meta-analyses. A last search was made just before the final analysis in order to include the most recently published studies.

### 2.2. Study Selection

Studies on the acute effect of exercise on AS were included in the meta-analysis. The inclusion criteria were the following: (i) population: healthy subjects aged between 20 and 50 years; (ii) intervention: type of exercise (interval training, aerobic exercise, resistance training, stretching); (iii) outcome: AS measured by PWv; (iv) comparison: pre–post exercise intervention.

We excluded (i) reports on the effect of exercise measured 24 h after the intervention; (ii) review articles, editorials, or case reports; (iii) studies including interventions combining exercise with dietary or pharmacological treatments; (iv) reports of two exercise sessions in a study less than 48 h apart; (v) articles that were not written in English or Spanish.

The selection of the studies was performed independently by two researchers (A.S.-L. and I.C.-R.). When agreement was not reached, a third reviewer (C.Á.-B.) was consulted.

### 2.3. Data Extraction and Risk of Bias Assessment

The main characteristics of the included studies are summarized in Table 1, which includes information on (1) reference (first author and year of publication), (2) country in which the study data were collected, (3) study design (crossover randomized control trials, non-randomized clinical trial), (4) population characteristics (sample size, mean age), (5) outcome: PWv (type of PWv (aortic PWv (a-PWv), brachial-ankle PWv (ba-PWv), brachial-radial PWv (br-PWv), carotid-dorsalis pedis PWv (cd-PWv), carotid-femoral PWv (cf-PWv), carotid-radial PWv (cr-PWv), femoral-ankle PWv (fa-PWv), femoral-dorsalis pedis PWv (fd-PWv)), basal PWv values, assessment time points, and method used), and (6) exercise intervention (interval training, aerobic exercise, resistance training, or stretching).

The quality of crossover randomized control trials was assessed using the Cochrane Collaboration’s tool for assessing risk of bias (Rob2) [24]. This tool evaluates the risk of bias according to six domains: selection bias, performance bias, detection bias, attrition bias, reporting bias, and other biases. The overall bias is considered “low risk of bias” if all domains are classified as “low risk”, “some concerns” if there is at least one domain rated as “some concern”, and “high risk of bias” if there is at least one domain rated as “high risk” or several domains rated as “some concerns”.

For non-randomized clinical trials, the risk of bias in non-randomized studies of interventions (ROBINS-I) tool [25] was used. This tool includes the assessment of bias in seven domains: confounding, selection of the study participants, measurement of interventions, deviations from intended interventions, missing data, measurement of outcomes, and bias in the selection of reported results. The overall risk of bias is rated as “low risk” if all domains are classified as “low risk”, “moderate risk” if all domains are classified as “low risk” or “moderate risk”, “serious risk” if there is at least one domain rated as “serious risk”, “critical risk” if there is at least one domain rated as “critical risk”, and “no information” if there is no clear indication that the study is at serious or critical risk of bias and if there is a lack of information in one or more key domains of bias.

Data extraction and quality assessment were conducted by two independent reviewers (A.S.-L., I.C.-R.). Disagreements were solved by consensus or with the intervention of a third researcher (C.Á.-B.).

### 2.4. Data Synthesis and Statistical Analysis

The DerSimonian and Laird method [55] was used to calculate a pooled effect size (ES) estimate and the respective 95% confidence intervals (CI) for central and peripheral PWv values. For central AS values, we included both a-PWv and cf-PWv, and for peripheral AS values we included ba-PWv, br-PWv, cd-PWv, cr-PWv, fa-PWv and fd-PWv. Considering the different PWv measurement times after intervention, the meta-analysis was performed by time periods (i.e., Period 1 (0–14 min post), Period 2 (15–29 min post), Period 3 (30–59 min post), and Period 4 (60 min–24 h post)). In addition, a meta-analysis was performed for carotid–femoral PWv (cf-PWv) separately from the other PWv measurements by time periods. Pooled estimates of the mean change difference (MD) for the acute effect of the exercise on cf-PWv (meters/second) were calculated.

The heterogeneity of the results among the studies was evaluated using the *I*^2^ statistic, which ranges from 0 to 100%. According to the *I*^2^ value [56], heterogeneity was considered not important (0 to 40%), moderate (30 to 60%), substantial (50 to 90%), or considerable (75 to 100%). The corresponding *p*-values were also taken into account.

A sensitivity analysis (systematic reanalysis while removing studies one at a time) was conducted to assess the robustness of the summary estimates. The results of sensitivity analyses were considered significant when the resulting estimates were modified beyond the CI of the original summary estimate. Subgroup analyses were performed according to exercise type (interval training, aerobic exercise, resistance training, stretching). Additionally, a subgroups analysis was conducted according to the age of participants (young participants: <30 years, and middle-aged participants: ≥30 years). A random-effects meta-regression analysis was performed to determine whether mean age, as a continuous variable, modified the acute effect of exercise on AS.

Finally, publication bias was assessed through Egger’s regression asymmetry test [57]. A level of <0.10 was used to determine whether publication bias might be present.

The statistical analyses were performed using Stata/SE software, version 15 (StataCorp, College Station, TX, USA).

## 3. Results

### 3.1. Systematic Review

**Figure 1:**
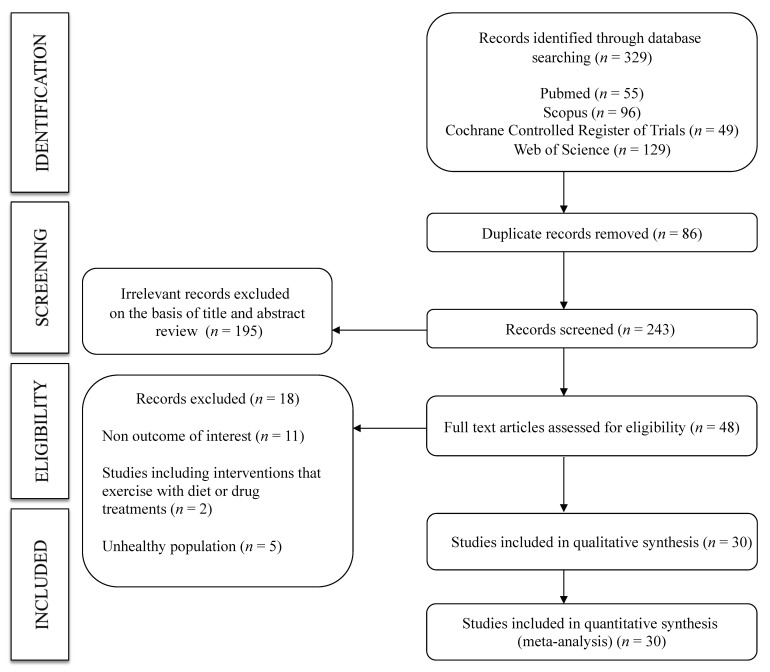
Flowchart: search strategy.

A total of 30 studies [17,26,27,28,29,30,31,32,33,34,35,36,37,38,39,40,41,42,43,44,45,46,47,48,49,50,51,52,53,54] were included in this systematic review and meta-analysis (Figure 1). All were non-randomized trials, except for six crossover randomized trials. The studies were published between 1997 and 2019 and included a total of 763 healthy adults (aged 20 to 50 years) assigned to an exercise intervention. Regarding the type of PWv measured, 22 studies included cf-PWv, 8 included fd-PWv, and the remaining studies included other measures, such as cr-PWv, fa-PWv, a-PWv, ba-PWv, br-PWv, or cd-PWv. The assessment time points included ranged from 0 min to 24 h after exercise. The most frequent type of exercise was aerobic exercise (22 studies), followed by interval training (five studies), resistance training (five studies) and stretching (three studies). Furthermore, seven studies reported on two exercise interventions (interval training—aerobic exercise: four studies, aerobic exercise—aerobic exercise: two studies, aerobic exercise—resistance training: 1 study). The characteristics of the included studies are shown in Table 1.

### 3.2. Risk of Bias

The overall risk of bias in the RCTs showed some concerns in all the included studies. Regarding the specific domains, in missing outcome data and in the selection of the reported results, 100.0% of the studies were scored as low bias; in the randomization process and measuring of the outcome, 100.0% were rated as some concerns; finally, in the deviations from intentional interventions domain, 33.3 and 66.7% of the studies showed a low risk of bias and some concerns, respectively (Figures S1 and S2).

The overall risk of bias from non-randomized trials showed some moderate or serious risk of bias in the included studies. Regarding the specific domains, the risk was rated as follows: For confounding, 72.4% of the studies were rated as moderate; for selection of study participants, 78.2% were rated as low; for the measurement of interventions domain, 87.2% were rated as moderate; for deviations from intended interventions, 94.7% were rated as moderate. In the remaining three domains (bias due to missing data, bias in the measurement of outcomes, and bias in the selection of reported results), most studies showed a moderate risk of bias (76.0, 100.0, and 80.4, respectively) (Figure S3).

### 3.3. Meta-Analysis

**Figure 2:**
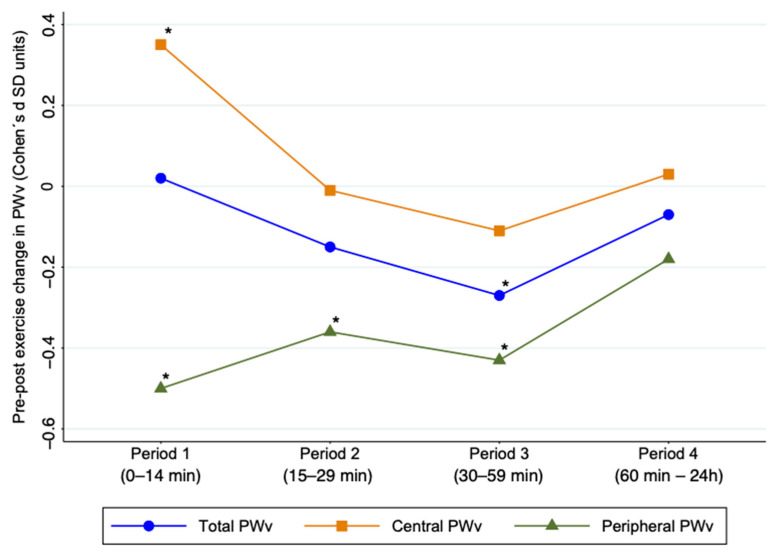
Acute effect of exercise on pulse wave velocity by time period after exercise. PWv: pulse wave velocity; SD: standard deviation * Values *p* < 0.05 were considered significant.

Figure 2 shows the acute effects of exercise on total, central, and peripheral PWv. Central PWv showed a significant increase in Period 1 after exercise (ES: 0.35; 95% CI: 0.04, 0.65) and peripheral PWv showed a significant decrease in Periods 1, 2, and 3 after exercise (ES: −0.50; 95% CI: −0.68, −0.32; ES: −0.36; 95% CI: −0.65, −0.08; ES: −0.43; 95% CI: −0.67, −0.19, respectively). Heterogeneity for central PWv estimation was substantial (*I*^2^ = 83.7%; *p* < 0.001), and for peripheral PWv, was considered not important in all cases (*I*^2^ = 0.0%, *p* = 0.87; *I*^2^ = 37.2, *p* = 0.121; *I*^2^ = 52.3%, *p* = 0.009).

In addition, the meta-analysis performed for cf-PWv showed not significant results. The pooled MD estimates for cf-PWv showed a significant increase in total m/sec in Period 1 (MD: 0.44; 95% CI: 0.09, 0.79) (Figure S5) with substantial heterogeneity (*I*^2^ = 86.9%; *p* < 0.001).

### 3.4. Sensitivity Analysis

The pooled ES estimate was not significantly modified (in magnitude or direction) when individual study data were removed from the analysis one at a time.

### 3.5. Subgroup Analyses and Meta-Regression

**Figure 3:**
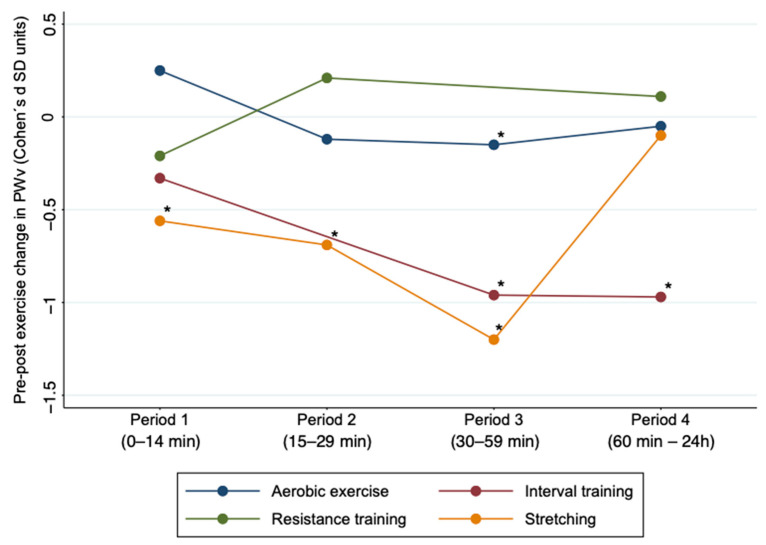
Acute effect of different types of exercise on pulse wave velocity by time period after exercise. PWv: pulse wave velocity; SD: standard deviation. * Values *p* < 0.05 were considered significant.

Subgroup analyses based on the type of exercise (interval training, aerobic exercise, resistance training, stretching) showed that aerobic exercise in Period 3 (ES: −0.15; 95% CI: −0.27, −0.02) was effective in reducing AS. Also, interval training showed significant results in Periods 3 and 4 (ES: −0.96; 95% CI: −1.72, −0.19; ES: −0.97; 95% CI: −1.75, −0.19), and stretching showed significant results in Periods 1, 2 and 3 (ES: −0.56: 95% CI: −0.85, −0.27; ES: −0.69; 95% CI: −1.1, −0.22; ES: −1.2; 95% CI: −1.76, −0.65) (Figure 3 and Supplementary Materials Table S2). For subgroup analyses based on the age of the participants, the young participants showed significant results in Period 3 (ES: −0.33; 95% CI: −0.5, −0.17) and middle-aged participants showed significant results in Period 1 (ES: −0.24; 95% CI: −0.48, −0.01) (Table S3).

The random-effects meta-regression models showed that the mean age in period 3 could influence the pooled estimates of the effect on AS (*p* = 0.023) (Table S4).

### 3.6. Publication Bias

Evidence of publication bias was observed through Egger’s test for all types of exercise (*p* = 0.006). Also, publication bias was found in the subgroup of resistance training (*p* = 0.019) and stretching (*p* = 0.044).

## 4. Discussion

This systematic review and meta-analysis provides an overview of the evidence analyzing the acute effects of exercise interventions on AS in healthy subjects. Our findings show that the peripheral PWV values present the highest acute effect from exercise, showing a significant decrease immediately after exercise, lasting 24 h. Furthermore, a decrease in central PWv was observed between 30 and 59 min after exercise. Additionally, aerobic exercise, interval training and stretching decreased PWv values, mainly between 30 and 59 min after exercise.

Some mechanisms have been proposed to explain the improvement of AS probably due to the functional but not structural adaptation of the vascular network to exercise [12,58,59]. This effect is a complex result of different physiological reactions to exercise: increased blood pressure and cardiac output, decreased peripheral resistance and vascular smooth muscle tone with vasodilation and increased arterial compliance in the muscular arteries. Some previous studies suggested that, under acute conditions, physical exercise increases blood flow and shear stress, leading to increased release of endothelial nitric oxide [60,61]; this elicits smooth muscle relaxation in response to constant stress caused by increased blood flow, explaining a sharp rise in arterial compliance and deceleration of PWv after exercise [62,63,64]. Additionally, during exercise, the arteries dilate the skeletal muscles by increasing blood flow through the release of vasodilatory signals (e.g., adenosine, lactate, K^+^, H^+^, CO_2_) from the surrounding active muscle [64]. Therefore, continuous exercise leads to an adaptive response of the arteries that includes increased vascular density and increased vasodilatory capacity, improving perfusion. This could be due to the adaptation of the endothelium to the interaction between recurrent hemodynamic tensions and the vasodilatory stimuli of exercise [65].

The elasticity of the large arteries is a consequence of the elastin/collagen ratio, which gradually decreases towards the periphery [66]. This alters the tone of the peripheral arteries and is thus able to modify the speed of movement of the pressure wave along their length [67]. As our results show, immediately after exercise the peripheral PWV was decreased. In contrast, the central PWv was increased immediately after exercise and decreased within 30 min after exercise. This effect could be mediated by the alteration of vascular muscle with exercise, possibly induced by components such as endothelium-derived hyperpolarizing factor [68]. The peripheral PWv after exercise shows vessel dilation due to increased shear stress, decreased vessel wall thickness, and reduced vasomotor tone [69]. In fact, our findings in the peripheral PWv could be bigger than the results of the present study are showing because the data of the mixed central and peripheral PWv have been considered as data of the peripheral PWv and, as a consequence, in mixed (central and peripheral) PWv, the central PWv could have blunted the effect of the peripheral PWv.

Furthermore, the acute effect of exercise on PWv may be mediated by the effect on blood pressure. Blood pressure and PWv are closely related, so much so that it is still debated whether the former represents a cause or a consequence of the latter [70]. During exercise there is an increase in blood pressure, probably due to the action of the sympathetic nervous system [69]. Immediately after exercise, there is hypotension in the systolic and diastolic blood pressure that lasts up to two hours [69,71]. A total of twenty-four hours after exercise, there is a slight increase in blood pressure compared to the immediate effect after exercise [71]. These variations in blood pressure match with the changes that occur at the peripheral level in PWv after exercise in our results. The relationship between peripheral PWv and blood pressure could be due to the elasticity of the large arteries, which gradually decreases towards the periphery [66]. Exercise results in adaptation of the endothelium to the interaction between hemodynamic tensions and vasodilator stimuli [65].

There is evidence that acute aerobic exercise is effective in decreasing AS [13], which is associated with a lower risk of CVD and mortality [58,59]. The decrease in PWv after acute aerobic exercise of moderate intensity for 30 min could be effective in acute pathological disorders, such as to remedy the postprandial impairments of arterial stiffness produced by a diet high in carbohydrates and lipid [12,72,73]. Notwithstanding, acute aerobic exercise may have the opposite effect and cause acute pathological disorders, such as rupture of lipid-rich plates that are prone to rupture. Therefore, the mechanisms behind acute exercise effects, which could influence the improvement of PWv, are multiple and complex. In acute situations, some studies suggest that aerobic exercise increases blood flow by stimulating the production of endothelial nitric oxide [60,61]. Other mechanisms involved could be vasodilation mediators, decreased release of vasoconstriction factors (such as endothelin-1 or angiotensin-2), or vascular modifications [45]. Our findings, contrary to those of a recent meta-analysis [11], show a significant decrease in PWv from 30 to 59 min after acute aerobic exercise. This discrepancy in the results is due to the fact that, in our study, we performed the analysis by time periods, and we observed when the acute exercise was effective; however, the meta-analysis named above performed analyses according to the total acute effect of the exercises included [11].

There are some limitations that should be acknowledged. First, most of the included clinical trials are non-randomized studies, which implies that our results should be interpreted with caution. Second, the clinical trials included are generally of medium quality. Third, there was evidence of publication bias by Egger’s test, and unpublished results could modify the findings of this meta-analysis. Fourth, interpretation of the results is limited by the diversity of the exercise programs. Fifth, the lack of a control group should be remedied in future studies to facilitate interpretation and improve the generalizability of AS findings to cardiovascular health. Sixth, studies using other measures of arterial stiffness than PWv, like local arterial stiffness, have not been included in this systematic review and meta-analysis. Seventh, the scarcity of studies examining the effect of some types of exercise should be highlighted. Therefore, high-quality RCTs testing different exercise interventions in populations with different characteristics are needed to further investigate the acute effects of exercise interventions on AS.

## 5. Conclusions

Although numerous studies have shown that exercise is effective in reducing AS in the long term, our results show an acute effect on peripheral PWv in the following 24 h, after which time the baseline values are recovered. Additionally, aerobic exercise, interval training and stretching seems to show a greater acute effect on PWv reduction. These findings are of clinical importance showing that the acute effect of exercise on AS is probably a functional, rather than a structural, effect. However, since functional and structural changes are two interrelated and inseparable aspects of an integrated process of vessel adaptation, the acute effect of regularly applied exercise could result in a structural effect. Our results should be taken with caution, from the point of view of clinical practice, since for a change to occur at the vascular level it is necessary to prescribe exercise by defining the number of weekly sessions, their intensity, and their duration, and to include it in the lifestyles of the population.

## Figures and Tables

**Table 1:** Characteristics of studies included in the meta-analysis.

Reference	Country	Study Design	Population Characteristics		Outcome (Arterial Stiffness: PWv)				Exercise Intervention
			Sample Size (*n*)	Age (Years)	Type of PWv	Basal PWv (m/s)	Assessment Time Points	Method Used	
Kingwell et al., 1997 [26]	Australia	Crossover	12	24.0 ± 6.0	cf-PWv fd-PWv	6.2 ± 1.4 8.3 ± 1.0	Baseline and 30 min post	Doppler flow velocimeter	AE
Naka et al., 2003 [27]	UK	Non randomized CT	25	31.0 ± 6.0	ba-PWv	7.6 ± 1.1	Baseline and 60 min post	QVL SciMed	AE
Sugawara et al., 2003 [28]	Japan	Non randomized CT	18	24.0 ± 4.2	fa-PWv	9.1 ± 1.3	Baseline and 2 min post	Form PWV/ABI	AE
Heffernan et al., 2006 [29]	USAs	Non randomized CT	13	21.5 ± 2.5	cf-PWv fd-PWv	6.1 ± 1.1 7.9 ± 1.1	Baseline, 5 min and 25 min post	Task Force III	RT
Heffernan et al., 2007 (a) [30]	USAs	Non randomized CT	15	21.9 ± 2.3	cf-PWv	6.4 ± 1.6	Baseline, 10 min, 20 min and 30 min post	SphygmoCor	AE
Heffernan et al., 2007 (b) [31]	USAs	Non randomized CT	14	27.9 ± 7.5	cf-PWv fd-PWv	7.2 ± 1.1 8.5 ± 0.8	Baseline and 20 min post	SphygmoCor	RT
Heffernan et al., 2007 (c) [32]	USAs	Non randomized CT	13	25.0 ± 2.5	cf-PWv fd-PWv	Session 1: 5.3 ± 0.6; Session 2: 4.8 ± 1.1 Session 1: 8.9 ± 0.9; Session 2: 8.7 ± 1.2	Baseline and 20 min post	Doppler flow velocimeter	Session 1: AE Session 2: RT
Barnes et al., 2010 [33]	USAs	Crossover	G1: 11 G2: 16	G1: 25.0 ± 3.3 G2: 24.0 ± 1.9	cf-PWv	G1: 7.9 ± 1.0 G2: 7.6 ± 1.0	Baseline, 90 min and 24 h post	Colin VP-2000	RT
Tordi et al., 2010 [34]	France	Non randomized CT	11	22.5 ± 2.3	cd-PWv	Session 1: 8.6 ± 0.3 Session 2: 8.7 ± 0.7	Baseline and 30 min post	Complior	Session 1: IT Session 2: AE
Vlachopoulos et al., 2010 [35]	Greece	Non randomized CT	20	36.0 ± 10.0	cf-PWv	6.7 ± 0.9	Baseline and 0 min post	Complior	AE
Doonan et al., 2011 [36]	Canada	Non randomized CT	G1: 53 G2: 24	G1: 23.1 ± 5.4 G2: 26.0 ± 6.7	cf-PWv	G1: 6.0 ± 0.7 G2: 6.3 ± 1.0	Baseline, 2 min, 5 min, 10 min and 15min post	SphygmoCor	AE
Hull et al., 2011 [37]	UK	Non randomized CT	36	28.9 ± 9.0	cf-PWv	6.2 ± 1.6	Baseline and 0 min post	SphygmoCor	AE
McClean et al., 2011 [38]	Ireland	Crossover	8	22.9 ± 2.8	br-PWv	7.1 ± 1.8	Baseline and 0 min post	Sensor based PWV device	AE
Lane et al., 2012 [39]	USAs	Non randomized CT	G1: 31 G2: 31	G1: 24.7 ± 3.3 G2: 24.8 ± 3.3	cf-PWv fd-PWv	G1: 6.1 ± 1.2; G2: 5.5 ± 0.8 G1: 8.7 ± 1.7; G2: 8.1 ± 1.7	Baseline, 15 min and 30 min post	SphygmoCor	AE
Ranadive et al., 2012 [17]	USAs	Crossover	15	25.0 ± 5.0	cr-PWv cf-PWv fd-PWv	Session 1: 7.0 ± 0.8; Session 2: 6.6 ± 1.2 Session 1: 5.5 ± 1.2; Session 2: 5.6 ± 1.12 Session 1: 9.1 ± 1.9; Session 2: 8.7 ± 1.9	Baseline and 10 min post	SphygmoCor	Session 1: AE Session 2: AE
Burr et al., 2014 [40]	Canada	Non randomized CT	13	25.0 ± 6.0	cf-PWv	5.1 ± 0.6	Baseline, 10 min, 6 and 24 h post	SphygmoCor	AE
Jatoi et al., 2014 [41]	Saudi Arabia	Non randomized CT	23	23.0 ± 3.0	a-PWv	6.8 ± 0.9	Baseline, 5 min and 10 min post	Arteriograph	RT
Sun et al., 2014 [42]	China	Non randomized CT	G1: 32 G2: 30	G1: 28.0 ± 4.0 G2: 24.0 ± 4.0	cr-PWv cf-PWv fa-PWv	G1: 7.0 ± 1.0; G2: 7.1 ± 1.0 G1: 5.6 ± 1.1;G2: 5.6 ± 1.0 G1: 8.2 ± 1.2; G2: 8.9 ± 1.3	Baseline, 30 min and 60 min post	SphygmoCor	AE
Lefferts et al., 2015 [43]	USAs	Non randomized CT	12	22.0 ± 3.0	cf-PWv	G1: 5.0 ± 0.4 G2: 5.0 ± 0.4	Baseline and 15 min post	SphygmoCor	AE
Perdomo et al., 2016 [44]	USAs	Non randomized CT	30	23.8 ± 2.5	cf-PWv	6.1 ± 0.8	Baseline and 24 h post	Complior	AE
Siasos et al., 2016 [45]	Greece	Crossover	20	22.6 ± 3.3	cf-PWv fd-PWv	Session 1: 5.9 ± 0.7; Session 2: 5.9 ± 0.8 Session 1: 9.1 ± 1.1; Session 2: 9.2 ± 1.1	Baseline and 10 min post	SphygmoCor	Session 1: IT Session 2: AE
Yamato et el., 2016 [46]	Japan	Non randomized CT	26	21.0 ± 5.1	ba-PWv cf-PWv fa-PWv	NA NA NA	Baseline, 0, 15, 30 and 60 min post	Form PWV/ABI	S
Kingsley et al., 2017 [47]	USAs	Non randomized CT	9	22.0 ± 2.0	cf-PWv	5.3 ± 0.8	Baseline and 5 min post	SphygmoCor	IT
Yamato et al., 2017 [48]	Japan	Non randomized CT2	25	20.9 ± 1.5	fa-PWv	8.4 ± 0.8	Baseline, o and 15 min post	Form PWV/ABI	S
Logan et al., 2018 [49]	USAs	Non randomized CT	30	44.4 ± 10.9	cf-PWv	6.9 ± 1.5	Baseline and 5 min post	SphygmoCor	S
Okamoto et al., 2018 [50]	Japan	Non randomized CT	14	27.5 ± 3.7	fa-PWv	Session 1: 8.5 ± 0.9 Session 2: 8.6 ± 0.7	Baseline, 30 min and 60 min post	Form PWV/ABI	Session 1: IT Session 2: AE
Peres at al., 2018 [51]	France	Non randomized CT	15	48.5 ± 5.04	cr-PWv cf-PWv fd-PWv	Session 1: 8.2 ± 1.6; Session 2: 8.2 ± 1.9 Session 1: 9.3 ± 1.9; Session 2: 9.0 ± 1.6 Session 1: 9.1 ± 1.9; Session 2: 10.2 ± 2.7	Baseline and 5 min post	SPT-301	Session 1: IT Session 2: AE
Tomschi et al., 2018 [52]	Germany	Non randomized CT	G1: 21 G2: 20	G1: 23.3 ± 3.2 G2: 23.2 ± 2.3	a-PWv	IG1: 5.0 ± 0.4 IG2: 4.9 ± 0.4	Baseline and 0 min post	Mobil-O-Graph	AE
Perdomo et al., 2019 [53]	USAs	Crossover	15	45.4 ± 8.9	cr-PWv cf-PWv	Session 1: 8.5 ± 1.5; Session 2: 8.7 ± 1.4 Session 1: 6.8 ± 0.7; Session 2: 6.9 ± 0.76	Baseline, 30 min and 60 min post	Complior	Session 1: AE Session 2: AE
Schroeder et al., 2019 [54]	USAs	Non randomized CT	G1: 27 G2: 35	G1: 25.0 ± 4.0 G2: 24.0 ± 4.0	cf-PWv	G1: 5.8 ± 1.1 G2: 5.9 ± 1.1	Baseline, 15 min and 30 min post	SphygmoCor	AE

Data are shown as mean ± standard deviation (SD); AE: aerobic exercise; a-PWv: aortic pulse wave velocity; ba-PWv: brachial-ankle pulse wave velocity; br-PWv: brachial-radial pulse wave velocity; cd-PWv: carotid-dorsalis pedis pulse wave velocity; cf-PWv: carotid-femoral pulse wave velocity; cr-PWv: carotid-radial pulse wave velocity; CT: clinical trial; fa-PWv: femoral-ankle pulse wave velocity; fd-PWv: femoral-dorsalis pedis pulse wave velocity; G1: group 1: G2: group 2; IT: interval training; NA: not available; PWv: pulse wave velocity; RT: resistance training; S: stretching.

## Data Availability

No new data were created or analyzed in this study. Data sharing is not applicable to this article.

## Supplementary Materials

The following are available online at https://www.mdpi.com/2077-0383/10/2/291/s1, Table S1. Search strategy for the MEDLINE database, Table S2. Subgroup analysis according to the type of exercise in healthy subjects by time period after exercise, Table S3. Subgroup analysis according to age in healthy subjects by time period after exercise, Table S4. Meta-regression according to mean age in healthy subjects by time period after exercise, Figure S1. Quality assessment of included studies using the Cochrane Collaboration’s tool for assessing risk of bias (RoB2), Figure S2. Quality assessment using the Cochrane Collaboration’s tool for assessing risk of bias (RoB2), Figure S3. Quality assessment using the Risk of bias in non-randomized studies of interventions tool (ROBINS-I), Figure S4. Effect size for acute effect of exercise on carotid femoral pulse wave velocity by times period after exercise, Figure S5. Means difference for acute effect of exercise on carotid femoral pulse wave velocity by times period after exercise.
