# Supplementary material for: The Acute Effect of Exercise on Arterial Stiffness in Healthy Subjects: A Meta-Analysis
Source: J Clin Med. 2021 Jan 14;10(2):291. doi: 10.3390/jcm10020291 (PMC7831005; doi:10.3390/jcm10020291)
Supplement: Supplementary file 1 [file jcm-10-00291-s001.pdf]

Supplementary Files

Table S1. Search strategy for the MEDLINE database.

'Cardiovascular disease'<br>OR<br>'Cardiovascular risk'	AND	'Arterial stiffness'<br>OR<br>'Pulse wave velocity'<br>OR<br>'PWv'	AND	'Acute'<br>OR<br>'Acute effect'<br>OR<br>'Immediate effect'	AND	'Physical activity'<br>OR<br>'Exercise'<br>OR<br>'Training'	AND	'Healthy subjects'<br>OR<br>'Healthy participants'<br>OR<br>'Healthy people'<br>OR<br>'Healthy adults'
						'HIIT'<br>OR<br>'Interval training'<br>OR<br>'Intermittent exercise'		
						'Continuous exercise'<br>OR<br>'Aerobic exercise'<br>OR<br>'Endurance training'		
						'Resistance exercise'<br>OR<br>'Strength'		
						'Stretching'<br>OR<br>'Stretches'		

Table S2. Subgroup analysis according to the type of exercise in healthy subjects by time period after exercise.

	Period 1 (0–14 min Post)			Period 2 (15–29 min Post)			Period 3 (30–59 min Post)			Period 4 (60 min–24 h Post)		
	**No. Studies (Samples)**	**ES (95%CI)**	**I^2**	**No. Studies (Samples)**	**ES (95%CI)**	**I^2**	**No. Studies (Samples)**	**ES (95%CI)**	**I^2**	**No. Studies (Samples)**	**ES (95%CI)**	**I^2**
Interval training	3 (6)	−0.33 (−0.7, 0.05)	39.6%	-	-	-	2 (2)	**−0.96 (−1.72, −0.19)**	39.4%	1 (1)	**−0.97 (−1.75, −0.19)**	–
Aerobic exercise	11 (25)	0.25 (−0.07, 0.57)	84.6%	6 (13)	−0.12 (−0.3, 0.05)	19.4%	8 (21)	**−0.15 (−0.27, −0.02)**	0.0%	6 (15)	-0.05 (−0.22, 0.11)	16.3%
Resistance training	2 (4)	−0.21 (−0.53, 0.12)	0.0%	3 (6)	0.21 (−0.1, 0.52)	0.0%	-	-	-	1 (4)	0.11 (−0.26, 0.49)	0.0%
Stretching	**3 (5)**	**−0.56 (−0.85, −0.27)**	**0.0%**	2 (4)	**−0.69 (−1.1, −0.22)**	23.1%	1 (3)	**−1.2 (−1.76, −0.65)**	24.4%	1 (3)	−0.1 (−0.54, 0.34)	0.0%

Table S3. Subgroup analysis according to age in healthy subjects by time period after exercise.

	Period 1 (0–14 min Post)			Period 2 (15–29 min Post)			Period 3 (30–59 min Post)			Period 4 (60 min–24 h Post)		
	**No. Studies (Samples)**	**ES (95%CI)**	**I^2**	**No. Studies (Samples)**	**ES (95%CI)**	**I^2**	**No. Studies (Samples)**	**ES (95%CI)**	**I^2**	**No. Studies (Samples)**	**ES (95%CI)**	**I^2**
Young participants (<30 years)	14 (32)	0.08 (−0.2, 0.37)	84.5%	10 (23)	−0.15 (−0.32, 0.02)	38.8 %	**9 (22)**	**−0.33 (−0.5, −0.17)**	**39.6%**	6 (18)	−0.11 (−0.26, 0.04)	4.2%
Middle-aged participants (≥30 years)	**3 (8)**	**−0.24 (−0.48, −0.01)**	**0.0%**	-	-	-	1 (4)	0.15 (−0.21, 0.5)	0.0%	2 (5)	0.16 (−0.16, 0.48)	0.0%

Table S4. Meta-regression according to mean age in healthy subjects by time period after exercise.

	**Coefficient**	**95%ICs**	***p* value**
**MEAN AGE**			
Period 1 (0–14 min post)	−0.01	−0.04, 0.02	0.386
Period 2 (15–29 min post)	0.07	−0.02, 0.16	0.137
Period 3 (30–59 min post)	**0.03**	**0.01, 0.05**	**0.023**
Period 4 (60 min–24 h post)	0.01	−0.01, 0.03	0.536

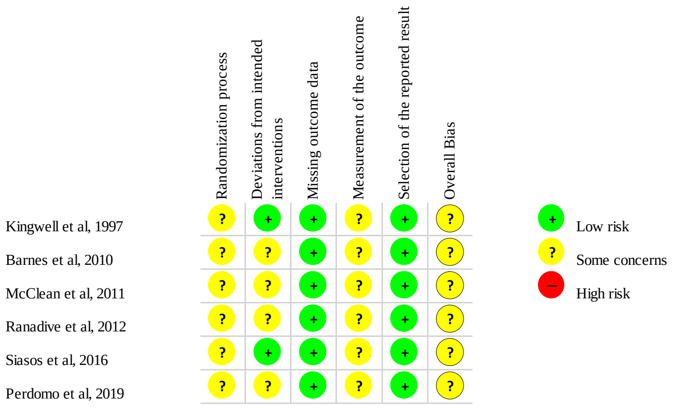

Figure S1. Quality assessment of included studies using the Cochrane Collaboration's tool for assessing risk of bias (RoB2).

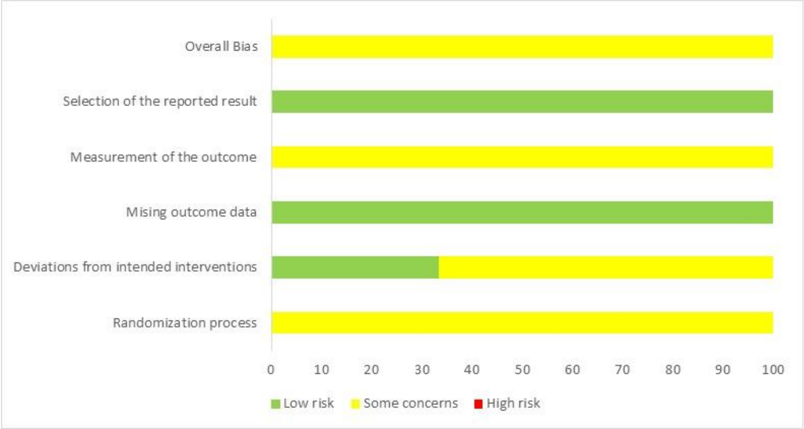

Figure S2. Quality assessment using the Cochrane Collaboration's tool for assessing risk of bias (RoB2).

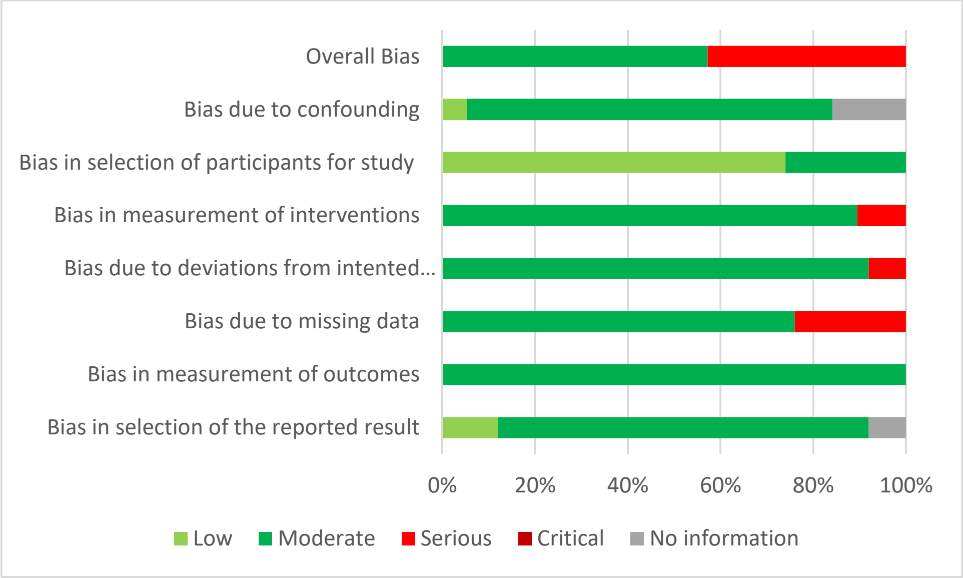

Figure S3. Quality assessment using the Risk of bias in non-randomized studies of interventions tool (ROBINS-I).

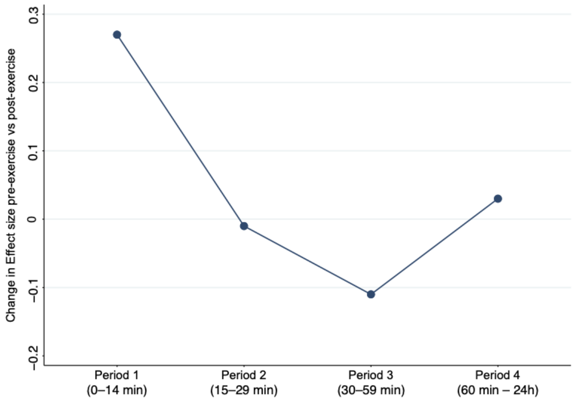

Figure S4. Effect size for acute effect of exercise on carotid femoral pulse wave velocity by times period after exercise. * Values $p < 0.05$ were considered significant.

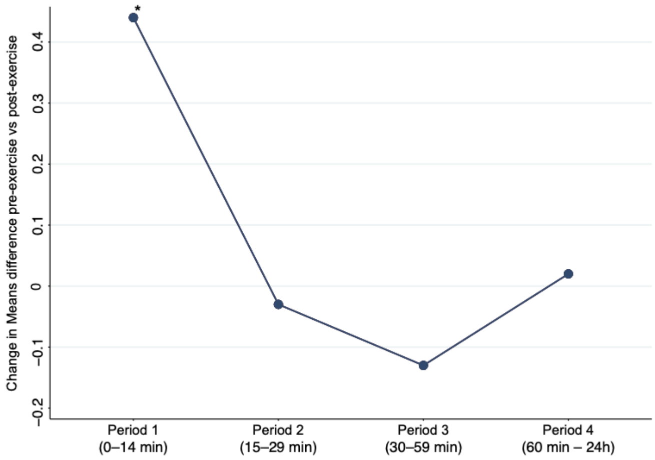

Figure S5. Means difference for acute effect of exercise on carotid femoral pulse wave velocity by times period after exercise. * Values $p < 0.05$ were considered significant
